# Relationship between Mitochondrial Quality Control Markers, Lower Extremity Tissue Composition, and Physical Performance in Physically Inactive Older Adults

**DOI:** 10.3390/cells12010183

**Published:** 2023-01-02

**Authors:** Anna Picca, Matthew Triolo, Stephanie E. Wohlgemuth, Matthew S. Martenson, Robert T. Mankowski, Stephen D. Anton, Emanuele Marzetti, Christiaan Leeuwenburgh, David A. Hood

**Affiliations:** 1 Fondazione Policlinico Universitario “A. Gemelli”, IRCCS, 00168 Rome, Italy; 2 Department of Medicine and Surgery, LUM University, 70100 Casamassima, Italy; 3 Muscle Health Research Centre, York University, Toronto, ON M3J 1P3, Canada; 4 School of Kinesiology and Health Science, York University, Toronto, ON M3J 1P3, Canada; 5 Department of Physiology and Aging, University of Florida, Gainesville, FL 32610, USA; 6 Department of Geriatrics and Orthopedics, Università Cattolica del Sacro Cuore, 00168 Rome, Italy

**Keywords:** aging, functional decline, intermuscular adipose tissue (IMAT), lysosomes, mitochondrial dysfunction, mitochondrion, muscle aging, physical performance, sarcopenia, Short Physical Performance Battery (SPPB)

## Abstract

Altered mitochondrial quality and function in muscle may be involved in age-related physical function decline. The role played by the autophagy–lysosome system, a major component of mitochondrial quality control (MQC), is incompletely understood. This study was undertaken to obtain initial indications on the relationship between autophagy, mitophagy, and lysosomal markers in muscle and measures of physical performance and lower extremity tissue composition in young and older adults. Twenty-three participants were enrolled, nine young (mean age: 24.3 ± 4.3 years) and 14 older adults (mean age: 77.9 ± 6.3 years). Lower extremity tissue composition was quantified volumetrically by magnetic resonance imaging and a tissue composition index was calculated as the ratio between muscle and intermuscular adipose tissue volume. Physical performance in older participants was assessed via the Short Physical Performance Battery (SPPB). Protein levels of the autophagy marker p62, the mitophagy mediator BCL2/adenovirus E1B 19 kDa protein-interacting protein 3 (BNIP3), the lysosomal markers transcription factor EB, vacuolar-type ATPase, and lysosomal-associated membrane protein 1 were measured by Western immunoblotting in vastus lateralis muscle biopsies. Older adults had smaller muscle volume and lower tissue composition index than young participants. The protein content of p62 and BNIP3 was higher in older adults. A negative correlation was detected between p62 and BNIP3 and the tissue composition index. p62 and BNIP3 were also related to the performance on the 5-time sit-to-stand test of the SPPB. Our results suggest that an altered expression of markers of the autophagy/mitophagy–lysosomal system is related to deterioration of lower extremity tissue composition and muscle dysfunction. Additional studies are needed to clarify the role of defective MQC in human muscle aging and identify novel biological targets for drug development.

## 1. Introduction

The maintenance of physical independence in late life is a public health priority and a key objective of major research agencies [1]. The physical function of an individual plays a critical role in maintaining independence. Measures of physical performance (e.g., walking speed, chair-stand tests) can predict the incidence of mobility disability and all-cause mortality, and can therefore be considered metrics of healthy aging [2,3,4,5,6,7]. Studies have shown that exercise training conveys multiple beneficial effects in older age, including preservation of mitochondrial mass, dynamics (mitochondrial fusion and fission), autophagy, and bioenergetics in muscle [8,9]. However, no drug is currently available to prevent or reverse the loss of physical function in advanced age, mainly because of insufficient understanding of its underlying mechanisms.

Of the various biological pathways possibly involved in the pathogenesis of age-related physical function decline, there is increasing evidence that alterations in mitochondrial quality and function in skeletal muscle [10,11,12,13,14], along with structural and functional muscle remodeling [15], may play a central role. A link between muscle iron handling, mitochondrial dysfunction, and systemic inflammation has recently been described in older adults [16]. In a later study, labile iron content was found to be increased in muscle of older adults with low physical performance, which was associated with altered expression of mitochondrial quality control (MQC) markers and greater mitochondrial DNA damage [17]. These findings suggest that loss of MQC efficiency may contribute to muscle dysfunction in advanced age.

The autophagy–lysosome system is a pivotal component of MQC. Through this system, proteins and organelles are tagged for disposal, engulfed into an autophagosome, and ultimately delivered to the lysosome for degradation [18]. Preclinical studies have shown that suppression of the autophagy–lysosome system leads to muscle atrophy, neuromuscular junction dysfunction, and muscle weakness [19,20,21,22,23]. Furthermore, the accumulation of nondegradable lysosomal content in the form of lipofuscin has been described in muscles of old mice and humans [24,25,26], which might be linked to lysosomal dysfunction and impairment of cellular quality control systems. In line with these findings, alterations in autophagy and mitophagy signaling were found to be associated with muscle atrophy and loss of physical function in older adults [14,21,27,28].

To better characterize the association between derangements in MQC and functional decline in advanced age, in the present study, we explored whether the expression of proteins pertaining to autophagy, mitophagy, and lysosomal pathways were altered as a function of age in both men and women. We also explored whether these alterations were linked to measures of lower extremity tissue composition and physical performance.

## 2. Materials and Methods

### 2.1. Participants

This study made use of muscle samples and data collected as part of a Developmental Project of the University of Florida Claude D. Pepper Older Americans Independence Center [13,29,30]. As previously reported, study participants were young (18 to 35 years) and old (70 years and older), physically inactive community-dwelling men and women [13,29,30]. The project protocol was approved by the University of Florida’s Institutional Review Board (IRB201300790), and all participants provided written informed consent prior to enrollment.

Eligibility criteria were chosen to minimize the confounding effect of comorbid conditions, medications, and lifestyle habits on biochemical measures and their relationship with physical performance [16,31]. Individuals were also excluded if they had contraindications to magnetic resonance imaging (MRI) acquisition.

### 2.2. Measurement of Lower Extremity Tissue Composition by Magnetic Resonance Imaging

Thigh and calf muscle and intermuscular adipose tissue (IMAT) of the dominant side were quantified volumetrically by T1-weighted 3-dimensional MRI [29,30]. Images were acquired on a 3.0-tesla magnet (Philips Medical Systems, Bothell, WA, USA) and were analyzed using the freely-available software MIPAV 1.3 (Medical Image Processing, Analysis and Visualization; Center for Information Technology, National Institutes of Health, Bethesda, MD, USA; http://mipav.cit.nih.gov, accessed on 10 October 2011), as previously described [29,30]. A lower extremity tissue composition index was subsequently calculated as the ratio between muscle volume and IMAT volume and used as an indicator of fatty infiltration in muscle [32].

### 2.3. Measurement of Physical Performance

Physical performance was assessed in old participants through the Short Physical Performance Battery (SPPB) [33]. The SPPB includes three tests: standing balance, gait speed at usual pace over a short track (e.g., 4 m), and the 5-time sit-to-stand test. Each of the three SPPB subtasks was scored from 0 (inability to do the test) to 4 (maximum performance), and the total score ranged from 0 to 12. The 4 m gait speed and the time needed to complete the 5-time sit-to-stand test were also recorded.

### 2.4. Collection of Muscle Biopsies

Muscle biopsies were obtained from the vastus lateralis of the dominant side by percutaneous needle biopsy under local anesthesia, as previously described [30]. Upon collection, muscle specimens were cleaned of any visible blood and fat, snap-frozen in liquid nitrogen, and stored at −80 °C until analysis.

### 2.5. Muscle Tissue Processing and Western Immunoblotting

Muscle samples were processed as described elsewhere [34]. Briefly, 15−20 mg of muscle tissue were pulverized under liquid nitrogen. Whole tissue extracts were obtained by dissolving the powder in 10× *w*/*v* Sakamoto buffer (20 mM HEPES, 2 mM ethylene glycol tetraacetic acid, 1% Triton X–100, 50% glycerol, 50 mM ß-glycerophosphate) supplemented with phosphatase (Sigma–Aldrich, St. Louis, MO, USA) and protease inhibitors (Roche, Mississauga, ON, Canada). Samples were rotated end-over-end for 1 h at 4 °C, sonicated on ice (3 s × 3 times), and centrifuged at 14,000× *g* for 15 min at 4 °C. The supernatant was collected and stored at −80 °C until analysis.

Protein concentration was determined using the Bradford method. Twenty–30 μg of total protein were separated via sodium dodecyl sulphate—polyacrylamide gel electrophoresis and transferred onto nitrocellulose membranes (Bio–Rad). Membranes were blocked at room temperature for 1 h in blocking solution (0.12% Tris–HCl, 0.585% NaCl, 0.1% Tween 20, 5% *w*/*v* skim milk, pH 7.5) and subsequently incubated overnight with primary antibodies at 4 °C (Table 1). The following day, membranes were washed in wash buffer (0.12% Tris–HCl, 0.585% NaCl, 0.1% Tween 20, pH 7.5) and incubated for 1 h at room temperature with the appropriate horseradish peroxidase-conjugated secondary antibody. Finally, the Clarity Western ECL Substrate solution (Bio–Rad) was applied and the chemiluminescent signal was captured with an Image Station 4000 MM Pro (Carestream, Concord, ON, Canada). Spot density of target bands were quantified by the ImageJ software (National Institutes of Health, Bethesda, MD, USA) and normalized to the density of glyceraldehyde-3-phosphate dehydrogenase (GAPDH, loading control).

### 2.6. Statistical Analysis

The normal distribution of data was verified via the Kolmogorov–Smirnov test. Comparisons between age groups for continuous variables were performed by Student’s *t* tests or Mann–Whitney U tests, as appropriate. Differences in the distribution of categorical variables between groups were assessed via χ^2^ statistics. Relationships between variables were explored by Spearman’s tests. All tests were two-sided, with statistical significance set at *p* < 0.05. All analyses were performed using the GraphPrism 5.03 software (GraphPad Software, Inc., San Diego, CA, USA).

## 3. Results

### 3.1. Characteristics of Study Participants

Twenty-three participants were included, nine young (four men and five women; mean age: 24.3 ± 4.3 years) and 14 older adults (eight men and six women; mean age: 77.9 ± 6.3 years). The main characteristics of study participants according to age groups are listed in Table 2. The two groups did not differ for sex distribution, body mass index values, or number of medications. The number of diseases was higher in the old participant group. In addition, old participants had significantly smaller lower extremity muscle volume, greater IMAT volume, and lower tissue composition index than their younger counterparts.

### 3.2. Measurement of Autophagy, Mitophagy, and Lysosomal Markers in Muscle Biopsies

Protein expression levels of the general autophagy marker p62, the mitophagy mediator BCL2/adenovirus E1B 19 kDa protein-interacting protein 3 (BNIP3), the lysosomal markers transcription factor EB (TFEB), vacuolar-type ATPase (vATPase), and lysosomal-associated membrane protein 1 (LAMP1) were measured in muscle samples of young and old participants.

**Figure 1:**
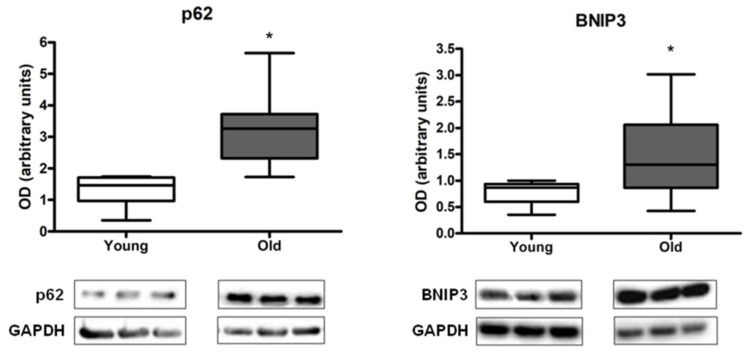
Protein content of p62 and BCL2/adenovirus E1B 19 kDa protein-interacting protein 3 (BNIP3) in muscle of young and old participants. Differences between age groups were analyzed by Mann–Whitney U statistics. Values are expressed as optical density (OD) and are reported in arbitrary units. Box plots represent median values (interquartile ranges). Representative blots are shown. Abbreviation: GAPDH, glyceraldehyde-3-phosphate dehydrogenase. * *p* < 0.05. young group (*n* = 7 for p62, *n* = 8 for BNIP3), old group (*n* = 14).

The protein content of p62 and BNIP3 was significantly higher in older than in young adults (Figure 1).

**Figure 2:**
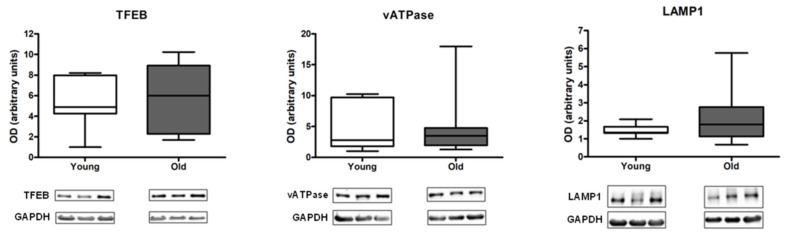
Protein content of lysosomal markers in muscle of young and old participants. Differences between age groups were analyzed by Mann–Whitney U statistics. Values are expressed as optical density (OD) and are reported in arbitrary units. Box plots represent median values (interquartile ranges). Representative blots are shown. Abbreviations: GAPDH, glyceraldehyde-3-phosphate dehydrogenase; LAMP1, lysosomal-associated membrane protein 1; TFEB, transcription factor EB; vATPase, vacuolar-type ATPase. young group (*n* = 7), old group (*n* = 14).

None of the assayed lysosomal markers showed statistically significant differences between young and old participants (Figure 2).

### 3.3. Relationship between Autophagy, Mitophagy, and Lysosomal Markers and Measures of Lower Extremity Tissue Composition and Physical Performance

**Figure 3:**
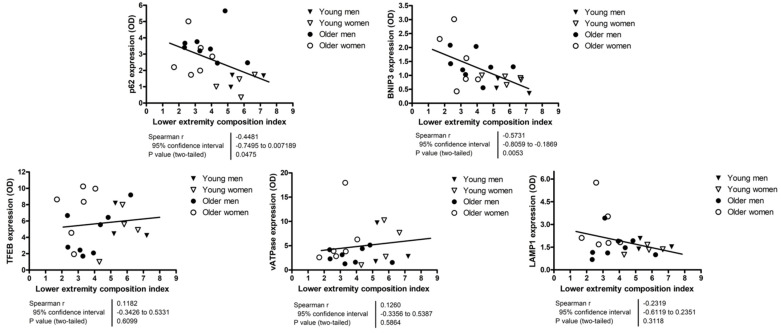
Correlation analyses between autophagy, mitophagy, and lysosomal markers and the lower extremity tissue composition index. Correlations were explored via Spearman’s statistics. The content of protein markers is expressed as optical density (OD) and is reported in arbitrary units. The lower extremity tissue composition index was calculated as the ratio between lower extremity muscle volume and intermuscular adipose tissue volume. Abbreviations: BNIP3, BCL2/adenovirus E1B 19 kDa protein-interacting protein 3; LAMP1, lysosomal-associated membrane protein 1; TFEB, transcription factor EB; vATPase, vacuolar-type ATPase.

Results of correlation analyses between autophagy, mitophagy and lysosomal markers and the lower extremity tissue composition index are shown in Figure 3. A statistically significant negative correlation was detected between both p62 (*r* = −0.4481; *p* = 0.0475) and BNIP3 (*r* = −0.5731; *p* = 0.0053) and the lower extremity tissue composition index. LAMP1 protein levels were negatively correlated with muscle volume (*r* = −0.4651; *p* = 0.0336) (Figure S1), while those of BNIP3 (*r* = 0.6454; *p* = 0.0012) and vATPase (*r* = −0.4857; *p* = 0.0256) were positively and negatively related with IMAT volume, respectively (Figure S2). 

**Figure 4:**
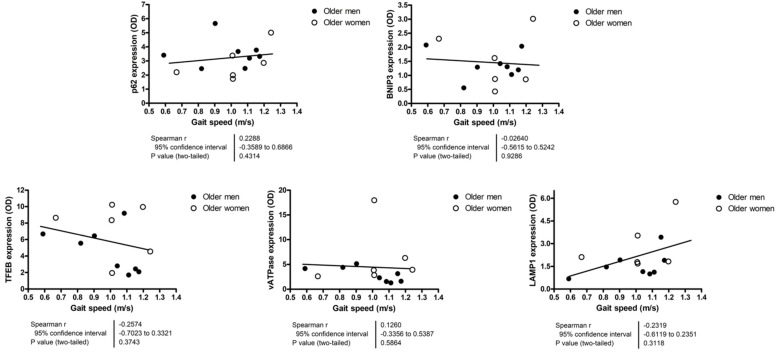
Correlation analyses between autophagy, mitophagy, and lysosomal markers and 4 m gait speed. Correlations were explored via Spearman’s statistics. The content of protein markers is expressed as optical density (OD) and is reported in arbitrary units. Abbreviations: BNIP3, BCL2/adenovirus E1B 19 kDa protein-interacting protein 3; LAMP1, lysosomal-associated membrane protein 1; TFEB, transcription factor EB; vATPase, vacuolar-type ATPase.

**Figure 5:**
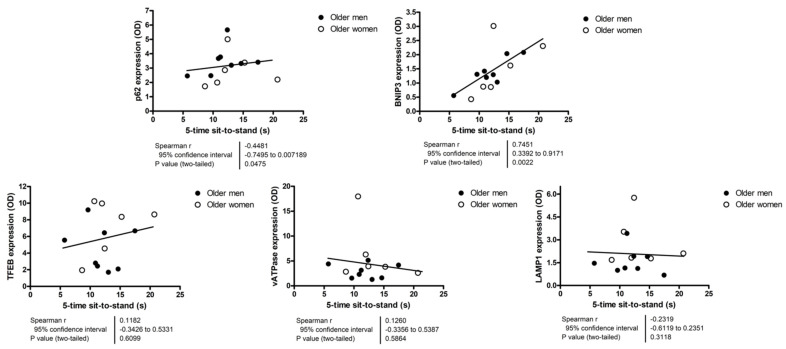
Correlation analyses between autophagy, mitophagy, and lysosomal markers and the 5-time sit-to-stand test completion time. Correlations were explored via Spearman’s statistics. The content of protein markers is expressed as optical density (OD) and is reported in arbitrary units. Abbreviations: BNIP3, BCL2/adenovirus E1B 19 kDa protein-interacting protein 3; LAMP1, lysosomal-associated membrane protein 1; TFEB, transcription factor EB; vATPase, vacuolar-type ATPase.

Because most participants scored 11 or 12 on the SPPB, correlation analyses were run using actual values of 4 m gait speed and time to complete the 5-time sit-to-stand test. None of the assayed markers showed significant correlation with the 4 m gait speed (Figure 4). The content of p62 and BNIP3 were positively correlated with the time needed to complete the 5-time sit-to-stand test (Figure 5).

## 4. Discussion

In recent years, the biological pathways underpinning age-associated muscle and functional decline have been actively investigated in both preclinical models and humans. MQC consists of coordinated processes including mitochondrial dynamics, mitochondrial biogenesis, and mitophagy [35]. Notably, mitochondrial dysfunction and mitophagy alterations are acknowledged as hallmarks of aging [36]. Furthermore, inefficient disposal of dysfunctional mitochondria may trigger pro-inflammatory pathways, thereby contributing to inflamm-aging and further amplifying mitochondrial deficiency [37,38,39,40,41,42]. Previous studies have provided initial evidence of the possible involvement of dysfunctional MQC in age-related muscle atrophy and loss of physical performance [13,14,43]. Lower protein expression levels of mitochondrial sirtuin 3, peroxisome proliferator-activated receptor-γ coactivator-1α, and the mitochondrial fusion protein optic atrophy 1 were found in skeletal muscle of physically inactive older adults relative to young controls [13]. Furthermore, reduced levels of the autophagy mediator microtubule-associated protein 1 light chain 3B were observed in skeletal muscle of older adults with sarcopenia compared with non-sarcopenic peers [27].

In the present investigation, we found that the expression of the autophagy protein p62 and the mitophagy-targeting protein BNIP3 was higher in older adults than in young controls (Figure 1). These findings suggest that mitophagy might be upregulated in the aged muscle, possibly as a compensatory response to cope with increased levels of mitochondrial dysfunction. This view is consistent with previous observations in old rodents showing higher basal mitophagy flux in skeletal muscle [24,44]. Our correlation analysis revealing a negative association between protein expression levels of p62 and BNIP3 and the lower extremity tissue composition index supports the hypothesis of a compensatory upregulation of mitophagy in the setting of deterioration of tissue composition (Figure 3).

The activity of the mitophagy–lysosome system is regulated, among other factors, by mitochondrial bioenergetics [24,34,44,45]. A decline in mitochondrial oxidative capacity can trigger TFEB expression and lead to upregulation of autophagic and lysosomal proteins [24,34,44,45]. This signaling pathway enables degradation of dysfunctional organelles through the autophagy–lysosomal system to maintain tissue homeostasis. In contrast to what has been observed in preclinical models [24,44], in our study, the protein content of the lysosomal mediators TFEB, vATPase, and LAMP1 was not different between age groups (Figure 2). This result, in conjunction with the increased expression of p62 and BNIP3 in old participants (Figure 1), suggests that MQC might be impaired in muscle with age. 

If MQC works properly, mitochondrial tagging and autophagosome formation are followed by mitochondrial clearance through the lysosomal system. The loss of coordination along this pathway results in mitophagy “clogging” and accumulation of intracellular waste [46]. Therefore, upregulation of upstream autophagy and mitophagy proteins in muscle of older adults not accompanied by increased expression of lysosomal markers may indicate greater autophagic signaling with no actual disposal of damaged mitochondria. This view is consistent with the identification of undigested lysosomal content in the form of lipofuscin granules in muscles of old rodents and humans [25,26]. It is noteworthy that lipofuscin-loaded cells often contain a high number of dysfunctional mitochondria with low membrane potential [47]. The accumulation of undegraded cellular waste and damaged mitochondria eventually impacts tissue homeostasis, resulting in the appearance of aging phenotypes, including muscle decay [47].

The negative correlation identified between protein levels of p62 and BNIP3 and the lower extremity tissue composition index (Figure 3) supports the idea of inefficient autophagy/mitophagy as a possible factor in muscle aging. In this regard, it should be noted that the extent of fat infiltration into muscle (myosteatosis) is proposed to be a more relevant indicator of age-related muscle dysfunction than the degree of muscle atrophy [48]. The hypothesis of MQC impairment as a possible mechanism in muscle dysfunction is further supported by the association found between greater protein expression of p62 and BNIP3 and worse performance on the 5-time sit-to-stand test (Figure 5). The latter is a well-known and easy-to-implement test to assess lower extremity muscle strength and power [49,50]. 

Although reporting novel findings, our study has limitations that deserve discussion. First, the cross-sectional design does not allow inferring on the time course of changes in the expression of autophagy, mitophagy, and lysosomal markers and those in lower extremity tissue composition or physical performance. Likewise, causality between those phenomena cannot be established. Second, available muscle specimens were frozen which did not allow mitochondrial functional assessments, measurement of mitophagy flux, or mitochondrial imaging to be conducted. Third, being the study a corollary of a two previous investigations [13,30], the limited amount of residual muscle tissue imposed assigning a certain level of priority among the autophagy, mitophagy, and lysosomal markers to be assayed. For the same reason, no markers of mitochondrial dynamics or biogenesis were measured, which prevented from obtaining a more comprehensive appraisal of MQC. Fourth, the sample size was small and included multiple comparisons. Therefore, some of the statistically significant findings may have been due to chance, while comparisons that did not reach statistical significance may have lacked statistical power. Fifth, although all participants were physically inactive, no objective measure of physical activity was obtained. Therefore, no information is available on the possible effects of varying levels of physical activity on the expression of autophagy, mitophagy, and lysosomal markers or indices of muscle quantity and lower extremity tissue composition. Finally, the content of GAPDH has been found to decrease in muscle with aging and its use as a loading control in Western immunoblotting experiments involving comparisons between young and older adults has been questioned [51]. However, in our study, protein levels of GAPDH did not differ between young and old participants.

Our study has also several strengths. Ours is among the few studies that have quantified biochemical markers pertaining to the autophagy–lysosomal system in human muscle. Our findings add to a growing literature pointing to dysfunctional MQC as a central mechanism in muscle loss during normal aging and across a wide spectrum of pathological conditions (e.g., sarcopenia, physical frailty, disuse muscle atrophy, chemotherapy-induced muscle wasting, cachexia) (reviewed in [52,53,54,55]). From this perspective, the assessment of MQC markers may serve the dual purpose of (1) helping untangle the events underpinning age-related functional decline and disease-associated muscle wasting, and (2) contribute to define the repertoire of geroscience markers to establish metrics of biological aging and efficacy endpoints for intervention studies. Circulating markers either as free molecules or associated with extracellular vesicles are actively investigated as proxy of biological processes in muscle [56,57]. Their validation could allow muscle-specific pathways to be explored noninvasively, which would facilitate the implementation of MQC assessments in research and clinical practice. Participants were recruited and characterized by established investigators in the fields of aging and muscle physiology. Tissue composition of lower extremities was carefully quantified via 3-dimensional MRI. Physical performance in older adults was assessed through the SPPB, one of the most used and informative functional tests in this age group.

## 5. Conclusions

Results of the present investigation suggest that the expression of selected markers of the autophagy/mitophagy–lysosomal system is altered in muscle of older adults, which might contribute to deterioration of lower extremity tissue composition and muscle dysfunction. Additional studies are needed to further elucidate the role of defective MQC in human muscle aging with the aim of identifying novel biological targets for drug development.

## Figures and Tables

**Table 1:** Primary Antibodies used for Western Immunoblotting.

Antibody (Manufacturer)	Catalogue No.	Dilution
BNIP3 (gift)	N/A	1:500
GAPDH (Abcam)	Ab8254	1:50,000
LAMP1 (Abcam)	Ab24170	1:500
p62 (Abcam)	Ab56416	1:1000
TFEB (Bethyl)	A303-673A	1:750
vATPase B1/2 (Santa Cruz)	Sc-55544	1:1000

Abbreviations: BNIP3, BCL2/adenovirus E1B 19 kDa protein-interacting protein 3; GAPDH, glyceraldehyde-3-phosphate dehydrogenase; LAMP1, lysosomal-associated membrane protein 1; TFEB, transcription factor EB; vATPase, vacuolar-type ATPase.

**Table 2:** Characteristics of Study Participants According to Age Groups.

Characteristic	Young Participants (*n* = 9)	Old Participants (*n* = 14)	*p* Value
Age (years), mean ± SD	24.3 ± 4.3	77.9 ± 6.3	<0.0001
Sex (female), n (%)	5 (55.5)	6 (43.0)	0.5518
BMI (kg/m^2^), mean ± SD	25.5 ± 4.4	27.1 ± 3.3	0.3289
Lower extremity muscle volume (cm^3^), mean ± SD	366.3 ± 74.8	276.2 ± 61.9	0.0058
Lower extremity IMAT volume (cm^3^), mean ± SD	61.8 ± 13.8	90.0 ± 25.9	0.0069
Lower extremity tissue composition index, mean ± SD	5.9 ± 0.9	3.4 ± 1.2	<0.0001
Number of diseases *, mean ± SD	0.7 ± 0.5	1.9 ± 1.0	0.0041
Number of medications ^#^, mean ± SD	2.7 ± 2.4	3.5 ± 2.9	0.4615
SPPB summary score, mean ± SD	---	10.2 ± 2.4	---
4 m gait speed (m/s), mean ± SD	---	1.00 ± 0.19	---
5-time sit-to-stand test (s), mean ± SD	---	12.5 ± 3.7	---

Abbreviations: BMI, body mass index; IMAT, intermuscular adipose tissue; SD, standard deviation; SPPB, Short Physical Performance Battery. * includes hypertension, coronary artery disease, prior stroke, peripheral vascular disease, diabetes, chronic obstructive pulmonary disease, and osteoarthritis. ^#^ includes prescription and over-the-counter medications, supplements, and pro re nata drugs.

## Data Availability

The data presented in this study are available on request from the corresponding author.

## Supplementary Materials

The following supporting information can be downloaded at: https://www.mdpi.com/article/10.3390/cells12010183/s1, Figure S1: Correlation analyses between autophagy, mitophagy, and lysosomal markers and lower extremity muscle volume; Figure S2: Correlation analyses between autophagy, mitophagy, and lysosomal markers and lower extremity intermuscular adipose tissue (IMAT) volume.
