# Supplementary material for: Relationship between Mitochondrial Quality Control Markers, Lower Extremity Tissue Composition, and Physical Performance in Physically Inactive Older Adults
Source: Cells. 2023 Jan 2;12(1):183. doi: 10.3390/cells12010183 (PMC9818256; doi:10.3390/cells12010183)
Supplement: Supplementary file 1 [file cells-12-00183-s001.zip › cells-2028147-supplementary.pdf]

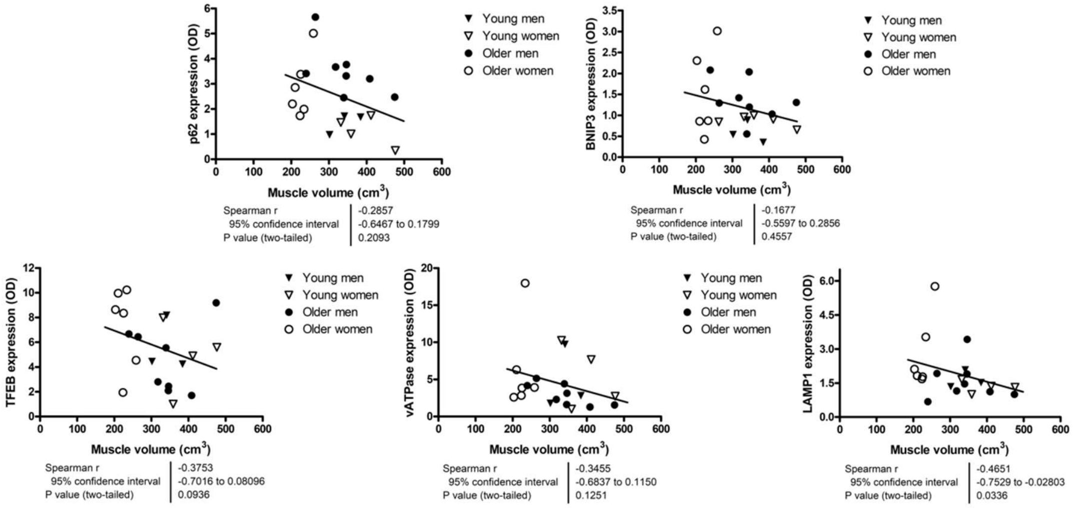

Figure S1. Correlation analyses between autophagy, mitophagy, and lysosomal markers and lower extremity muscle volume. Correlations were explored via Spearman's statistics. The content of protein markers is expressed as optical density (OD) and is reported in arbitrary units. Abbreviation: BNIP3, BCL2/adenovirus E1B 19 kDa protein-interacting protein 3; LAMP1, lysosomal-associated membrane protein 1; TFEB, transcription factor EB; vATPase, vacuolar-type ATPase.

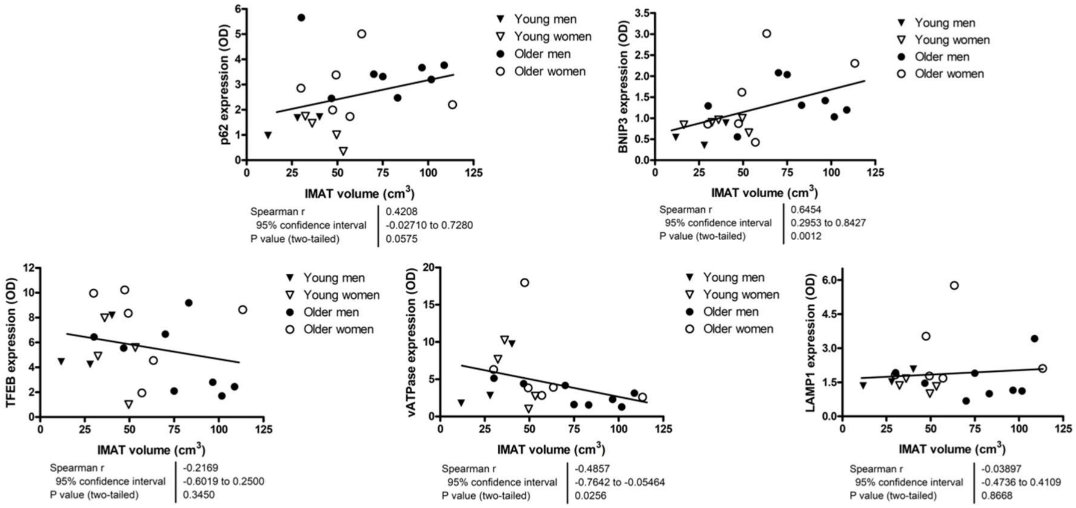

Figure S2. Correlation analyses between autophagy, mitophagy, and lysosomal markers and lower extremity intermuscular adipose tissue (IMAT) volume. Correlations were explored via Spearman's statistics. The content of protein markers is expressed as optical density (OD) and is reported in arbitrary units. Abbreviation: BNIP3, BCL2/adenovirus E1B 19 kDa protein-interacting protein 3; LAMP1, lysosomal-associated membrane protein 1; TFEB, transcription factor EB; vATPase, vacuolar-type ATPase.
